# Effectiveness of a new health care organization model in primary care for chronic cardiovascular disease patients based on a multifactorial intervention: the PROPRESE randomized controlled trial

**DOI:** 10.1186/1472-6963-13-293

**Published:** 2013-08-02

**Authors:** Domingo Orozco-Beltran, Esther Ruescas-Escolano, Ana Isabel Navarro-Palazón, Alberto Cordero, María Gaubert-Tortosa, Jorge Navarro-Perez, Concepción Carratalá-Munuera, Salvador Pertusa-Martínez, Enrique Soler-Bahilo, Francisco Brotons-Muntó, Jose Bort-Cubero, Miguel Angel Nuñez-Martinez, Vicente Bertomeu-Martinez, Vicente Francisco Gil-Guillen

**Affiliations:** 1 Unidad de docencia e investigación, Hospital Universitario de Sant Joan d’Alacant, Ctra. Nnal. 332 Alicante, Valencia s/n, Sant Joan d’Alacant Alicante 03550, Spain; 2 Servicio de Cardiología, Hospital Universitario de Sant Joan d’ Alacant, Ctra. Nnal. 332 Alicante, Valencia s/n, Sant Joan d’Alacant Alicante 03550, Spain; 3 CS Salvador Pau, c/ Salvador Pau, Nº 42, Valencia 46021, Spain; 4 Cátedra de Medicina de Familia. Departamento Medicina Clínica, Universidad Miguel Hernández, Ctra. Nnal. 332 Alicante-Valencia s/n, Sant Joan d’Alacant Alicante 03550, Spain; 5 CS Cabo Huertas, c/Arpón s/n, Alicante 03540, Spain; 6 CS Dolores Cano Royo, c/Martí l’Humá, 13, Vila-RealCastellón, Spain; 7 CS Carinyena c/Illes Columbretes, s/n 12540, Vila-Real, Castellon, Spain

**Keywords:** Health services research, Cardiovascular diseases, Primary care, Secondary prevention

## Abstract

**Background:**

To evaluate the effectiveness of a new multifactorial intervention to improve health care for chronic ischemic heart disease patients in primary care. The strategy has two components: a) organizational for the patient/professional relationship and b) training for professionals.

**Methods/design:**

Experimental study. Randomized clinical trial. Follow-up period: one year. Study setting: primary care, multicenter (15 health centers). For the intervention group 15 health centers are selected from those participating in ESCARVAL study. Once the center agreed to participate patients are randomly selected from the total amount of patients with ischemic heart disease registered in the electronic health records. For the control group a random sample of patients with ischemic heart disease is selected from all 72 health centers electronic records.

Intervention components: a) Organizational intervention on the patient/professional relationship. Centered on the Chronic Care Model, the Stanford Expert Patient Program and the Kaiser Permanente model: Teamwork, informed and active patient, decision making shared with the patient, recommendations based on clinical guidelines, single electronic medical history per patient that allows the use of indicators for risk monitoring and stratification. b) Formative strategy for professionals: 4 face-to-face training workshops (one every 3 months), monthly update clinical sessions, online tutorial by a cardiologist, availability through the intranet of the action protocol and related documents.

Measurements: Blood pressure, blood glucose, HbA1c, lipid profile and smoking. Frequent health care visits. Number of hospitalizations related to vascular disease. Therapeutic compliance. Drug use.

**Discussion:**

This study aims to evaluate the efficacy of a multifactorial intervention strategy involving patients with ischemic heart disease for the improvement of the degree of control of the cardiovascular risk factors and of the quality of life, number of visits, and number of hospitalizations.

**Trial registration:**

NCT01826929

## Background

The medical, health care, financial, personal and family burden of chronic disease is one of the main threats to the sustainability of the health system. At present, 70% of health care expenditure is used to treat chronic diseases [1]. Accordingly, health care systems are now moving towards more patient-centered models based on self-care and therapeutic education as ways of promoting the participation of the patients in their own treatment [2].

Cardiovascular disease (CVD) is one of the main chronic diseases, and is in fact the leading cause of death in the Spanish population. In 2007 it accounted for 124,126 deaths in Spain, representing 32% of all deaths [3]. In Spain, ischemic heart disease (IHD) causes most CVD deaths (30% overall; 37% in men and 24% in women). The hospital morbidity rate for IHD was 317 per 100,000 inhabitants (447 in men and 189 in women) [3]–[5].

Lifestyle changes (giving up smoking, a Mediterranean diet and exercise) have been shown to decrease cardiovascular morbidity and mortality in patients with IHD. In addition, much evidence now exists concerning pharmacological treatment aimed at the associated risk factors [5].

The latest European guidelines aim to increase the involvement of the primary care professionals in the implementation of the preventive activities for these patients. The guidelines emphasize a patient-centered approach, joint decision-making, and the importance of establishing realistic and feasible objectives, as tools to improve compliance with medication and lifestyle changes [6]–[8]. A recent Cochrane library review stresses the importance of primary care and the need to improve the organization of the health care services for the secondary prevention of IHD [9].

However, difficulties exist when incorporating the results of the various studies into clinical practice. Comparison of the results from the EUROASPIRE I to the EUROASPIRE III studies, in patients with IHD, shows that the prevalence of risk factors remains high: smoking hardly changed (20.3%, 21.2%, and 18.2%), obesity (body mass index ≤ 30) increased from 25% to 32.6% and 38%, and poorly controlled blood pressure (BP) (≥ 140/90 mmHg) changed very little (58.1%, 58.3%, and 60.9%). Only in the prevalence of hypercholesterolemia was an important decrease observed, from 94.5% to 76.7% and 46.2%. Concerning the use of drugs, between the EUROASPIRE I and the EUROASPIRE III studies [10] antiplatelet drugs rose from 80.8% to 93.2%, beta blockers from 56% to 85.5%, antihypertensive drugs from 84.5% to 96.8%, and lipid-lowering drugs from 32.2% to 88.8%. Although improvements have been noted, an important percentage of patients still exists in whom the control of risk factors could be improved.

Studies carried out in our area show that 54% of the patients with a history of myocardial infarction had hypercholesterolemia, 41% had high blood pressure, 11% were smokers, and 19% were obese; in addition, there was a clear underuse of medication [11].

Therapeutic educational interventions, such as the Chronic Care Model (CCM) that includes educational, organizational and community participation interventions; the Stanford Expert Patient Program (EPP); and the Kaiser Permanente model [12,13] have all shown their benefit in patients with a high CVD risk and their effect on clinical measures and health care use.

Based on these models, various initiatives to improve the secondary prevention of coronary disease patients have been undertaken in our area [14,15]. The ICAR study assessed the efficacy of an intensive secondary prevention program of coronary disease carried out in primary care. However, improvement was only found in blood pressure control and an increase in HDL-cholesterol concentrations [14]. The PREseAP study assessed the efficacy of an intervention carried out by nurses, but with no positive results [16,17]. Another intervention study also from primary care [18] found that admissions to hospital were significantly reduced, but no other clinical benefits were shown, possibly because of a ceiling effect related to improved management of the disease. Given these poor results, we designed a multifactorial intervention based on the CCM, integrating professionals from cardiology services and primary care, in an attempt to improve the degree of control of CVRF and reduce the number of hospital admissions.

### Main objective

To evaluate the effectiveness of a new multifactorial intervention in order to improve health care for chronic IHD patients in primary care. The strategy consisted of two components: a) organizational for the patient/professional relationship, and b) formative for the professionals.

### Specific objectives

Level of control/follow-up of the following variables: blood pressure, capillary blood glucose, HbA1c, LDL cholesterol, body mass index, therapeutic compliance, exercise, smoking, adherence to a Mediterranean diet, incidence of hospital admissions related to vascular disease, annual primary care visits (number of visits in one year) and drug use (antiplateled drugs, beta blockers, ACE inhibitors/ARA II, statins).

### Hypothesis

A multifactorial primary care intervention based on chronic models [12] can improve the level of control and reduce the number of hospital admissions in patients with IHD.

## Methods/design

### Study design

Experimental design. Type of study: open randomized clinical trial. Follow-up period: one year.

### Participants

Setting: Primary Health Care. Valencian Community (Spain).

15 Health centers: Alicante-Cabo-Huertas, Alicante-Campello, Alicante-Plà Hospital, Castellón-Dolores-Cano-Royo-Villareal, Castellón-La-Bobila-Villareal, Castellón-Cariñena-Villareal, Castellón-Nules, Castellón-Vall d’Uxo I and II; Valencia-S Pau, Valencia-R-Argentina, Valencia-Benimaclet and Valencia-Serreria-II.

Inclusion criteria: patients with a diagnosis of IHD of any site (ICD-10 codes from 410 to 414 inclusive); age from 30 to 80 years; signed written informed consent. Exclusion criteria: lack of consent; immobilized patients; patients with serious health problems or with a low life expectancy.

Data are collected from electronic health record system.

### Interventions

**Figure 1:**
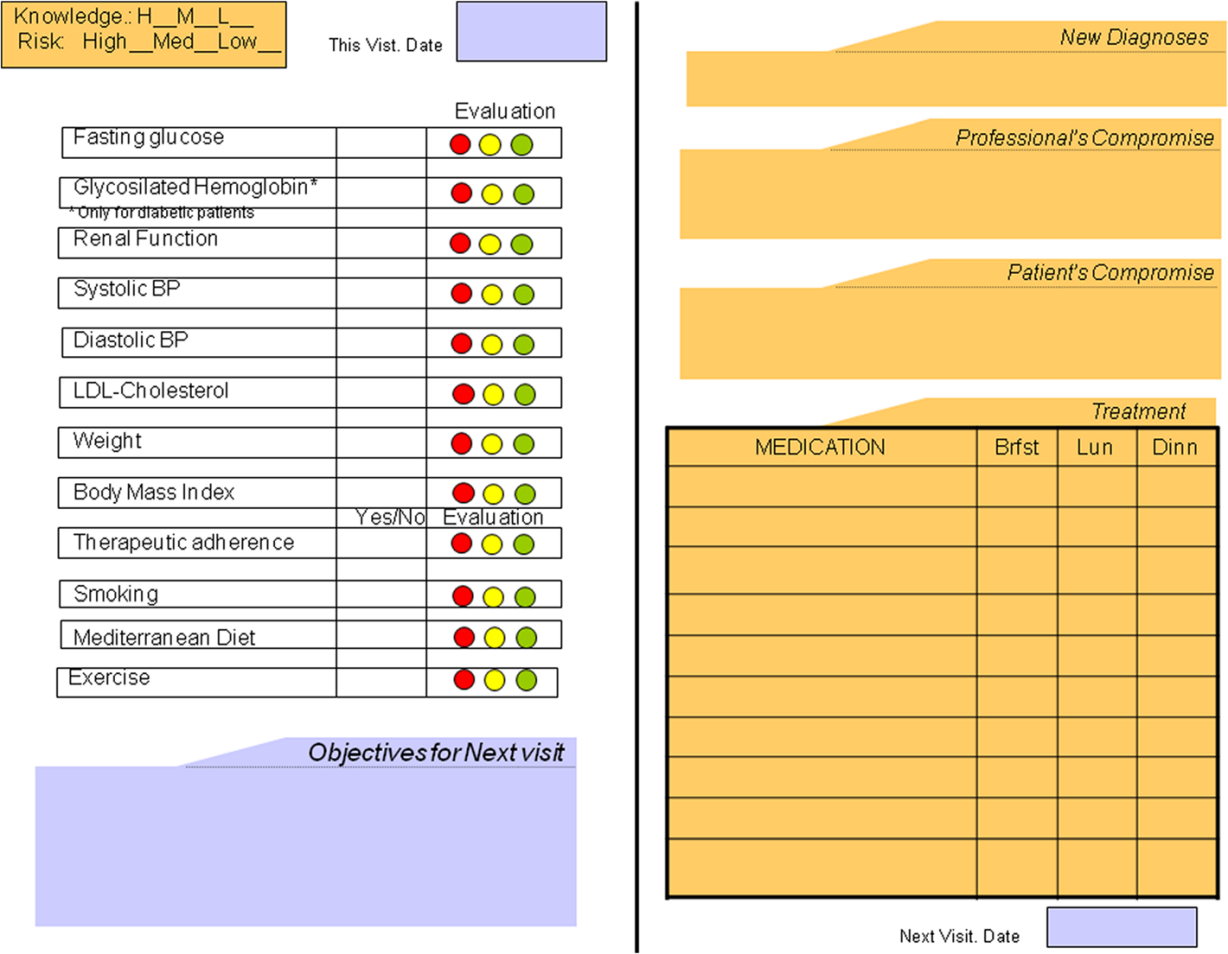
Personalized follow-up record.

Organized intervention strategy aimed at patients and professionals:

1. Organized intervention strategy:

a) Informed active patient. By means of therapeutic education of the patient according to the CCM recommendations and following the Stanford EPP model. To promote patient autonomy: self-measurement of blood pressure and blood glucose control. Use of recommendations for patients in a uniform format and content for all the professionals using the recommendation forms for patients with chronic diseases (hypertension, diabetes, IHD, obesity, smoking) available in the electronic medical history.

a) Shared decision making. Personalized control objectives. To seek ways of promoting the increased participation of chronic patients in their treatment and the sharing of decisions. Creation of a patient follow-up record showing the state of the different study variables. The achievement of the objectives is evaluated with a traffic light type graph identifying the degree of compliance in the colors green, amber or red. Personalized and agreed objectives are established for each patient and shown on the record (see Figure 1). The IHD patient care protocol of the SVMFiC (2010 update) is used [19], available via the Abucasis intranet. The criteria used to define good control of the variables (Table 1) are those of the Cardiovascular Disease Prevention Group of the Preventive Activity and Health Promotion Program (PAPPS) [5].

a) Appointment planning. Every three months, although variable depending on the patient, to provide time to evaluate the agreed changes. At nurse/patient visits to assess: diet, exercise and therapeutic compliance at 2 weekly sessions every 3 months (Tables 2 and 3).

a) Primary care doctor-nurse teamwork. Joint work with agreed aims in a basic primary care health unit.

a) Actions based on scientific evidence. Work following electronically available protocols validated by scientific societies integrated in the Valencian Medical Institute. Communication with the cardiology service, to facilitate the interconsultation between primary and secondary care.

a) To promote information systems through a single electronic history shared between primary and secondary care. Evaluation of indicators of program follow-up.

a) Formative intervention strategy for professionals:

a) Formative. Four face-to-face workshops (every 3 months); monthly clinical session on cardiovascular disease; online tutorials with the cardiology service to solve any doubts. Face-to-face tutorials with a team, reference doctor/nurse, in each health center.

a) Strategies to avoid clinical inertia: carry out therapeutic changes if there is poor control before 3 months.

**Table 1:** Therapeutic objectives

***Variable***	***Objective***
***Basal blood glucose***	< 126 mg/dl
***HbA1c***	< 7%;
***Blood pressure***	≤140/90 mmHg
***LDL cholesterol***	≤ 100 mg/dl
Therapeutic compliance*	80-110%
***Practice of exercise***	30 minutes per day > 3 days per week
***Mediterranean diet***	Validated survey.

(*)Pill count.

**Table 2:** Primary care visit timeline

***Visit timeline***												
***Follow-up months***	**1**	2	3	**4**	5	6	**7**	8	9	**10**	11	12
***Nurse***	**√**		√	**√**		√	**√**		√	**√**		√
***Physician***	**√**			**√**			**√**			**√**		

**Table 3:** Task distribution in the follow-up clinics

	***Risk factors***	***Physician***	***Nurse***
***1***	**Assess Abucasis register.**	++	++
***2***	**Establish personalized objectives.**	++	++
***3***	**Assess degree of control.**	++	++
***4***	**Assess therapeutic compliance.**	+	++
***5***	**Assess attitude towards each objective.**	++	++
***6***	**Agree interventions with the patient.**	++	++
***7***	**Adequate pharmacological treatment.**	++	-
***8***	**Individual intervention.**	++	++
***9***	**Team intervention.**	-	++
***10***	**Community intervention.**	-	++

### Outcome measures

Primary outcome measure: number of hospitalizations/cause. It is measured at the beginning and end of the study.

Secondary outcomes:

a) Level of control of CVRF: Blood pressure, LDL cholesterol, body mass index, basal blood glucose, HbA1c, therapeutic compliance (pill count), smoking. They are measured at each visit.

b) Healthy life habits: Exercise, mediterranean diet. They are measured at each visit.

c) Management: Number of annual primary care visits. It is measured at the end of the study.

To enhance the quality of measurements a training program is performed and there was one assessor for every three health centers.

### Sample size

The calculation of the sample size is carried out to compare means, with an alpha risk of 0.05 (95% confidence level) and a beta risk of 0.20 (80% power). For an estimated difference in SBP of 5 mmHg and a standard deviation of SBP of 2.5 mmHg, the number of persons necessary is 251 patients per group. Increased by 20% for possible losses, giving a total of 301 patients in each group.

### Randomization, allocation and implementation

Valencia is a Mediterranean Spanish Region with 5 million inhabitants. In 2007, 800 primary care physicians and nurses started the ESCARVAL primary prevention cohort study [20]. 72 Health centers (HC) participated including 50000 patients.

For the intervention group in the PROPRESE study 15 HC are selected from those participating in ESCARVAL. Once the center agreed to participate patients are randomly selected from the total amount of patients with IHD registered in the electronic health records.

For the control group a random sample of patients with IHD is selected from all 72 HC electronic records.

Patients are enrolled by their primary care physicians who will offer to participate to the randomly selected patient.

### Blinding

Blinding is not possible as the intervention is a multifactorial approach. That’s why the control group is selected after the intervention is made but for the same period of time.

### Statistical analysis

The results are expressed as frequencies and percentages for qualitative variables and as mean and standard deviation for the quantitative variables. For the statistical analysis, the study of categorical variables is carried out by the ×^2^ test and the comparison of the continuous variables between groups of patients by the Student t test and ANOVA. A multivariate analysis will be carried out to evaluate the effect of the intervention. The SPSS PC 15.0 program will be used.

The reference search was performed in the Medline database through PubMed. The MeSH terms used were: “Myocardial Ischaemia/prevention and control” [Mesh]” OR “Myocardial Ischaemia/epidemiology” [MeSH], OR “Coronary Artery Disease”[MeSH Terms] AND “Secondary prevention”. A second search included: “Patient Education” as Topic/methods OR “Patient Participation” OR “Self-Help Groups” OR “Program Evaluation” AND “Myocardial Ischaemia”.

In the Cochrane database we used the MeSH terms: Patient Education, Lifestyle.

The search was limited to publications in the last 5 years, only items with abstracts, studies in humans, and a publication type of Meta-Analysis and Systematic Review. In the Cochrane database we also used the search limit of the last 5 years.

### Follow-up

Table 4 shows the follow-up plan and patient data collection. Functions of the professionals participating in the study are described in Table 5.

**Table 4:** Follow-up plan and collection of patient data

			
**Enrolment allocation**	**Intervention group:**		
	15 HC selected from those participating in ESCARVAL-risk study.		
	350 patients randomly selected from the total amount of patients with IHD registered in the electronic health records.		
	Patients are enrolled by their primary care physicians. (Inclusion and exclusion criteria).		
	**Control group:**		
	A random sample of patients (350) with IHD selected from 72 HC electronic records (from ESCARVAL-risk study)		
**Intervention follow-up: 1 year**	Baseline data		
	Personalized follow-up record		
	Starting formative intervention strategy for professionals and patients:		
	**Intervention**	**Patients**	**Professionals**
	**First visit**	Evaluate monitoring of CVRF	Advice online by cardiologist
		Accord control objectives	Monthly updates
		Adherence control	Protocols, guides and bibliography reviews
		Therapeutic education	In-class training course
	**Second visit**	Evaluate monitoring of CVRF	Advice online by cardiologist
		Accord control objectives	Monthly updates
		Adherence control	Protocols, guides and bibliography reviews
		Therapeutic education	In-class training course
	**Third visit**	Evaluate monitoring of CVRF	Advice online by cardiologist
		Accord control objectives	Monthly updates
		Adherence control	Protocols, guides and bibliography reviews
		Therapeutic education	In-class training course
		Number of hospital admissions	
**Outcomes**	Statistical analysis. Loses to follow-up.		
	Presentation of results at participating HC.		
	Final report		

*HC*, Health Center.

**Table 5:** Functions of the professionals participating in the study

***Functions of each professional in the study***	
***Primary care physicians/nurses:***	- Identification of patients to include.
	- Inclusion and contact with patients.
	- Adaptation of therapeutic interventions.
	- Therapeutic compliance.
***Reference cardiologists and group of experts***	- Workshop teaching.
	- Online tutorials.
	- Selection of documents, guidelines and protocols.
***San Juan Alicante Department Research Unit***	- Analysis of the data and interpretation of the results
***Abucasis responsible***	- Facilitate the use of the electronic medical history through response to doubts or information about not very used resources.

**Figure 2:**
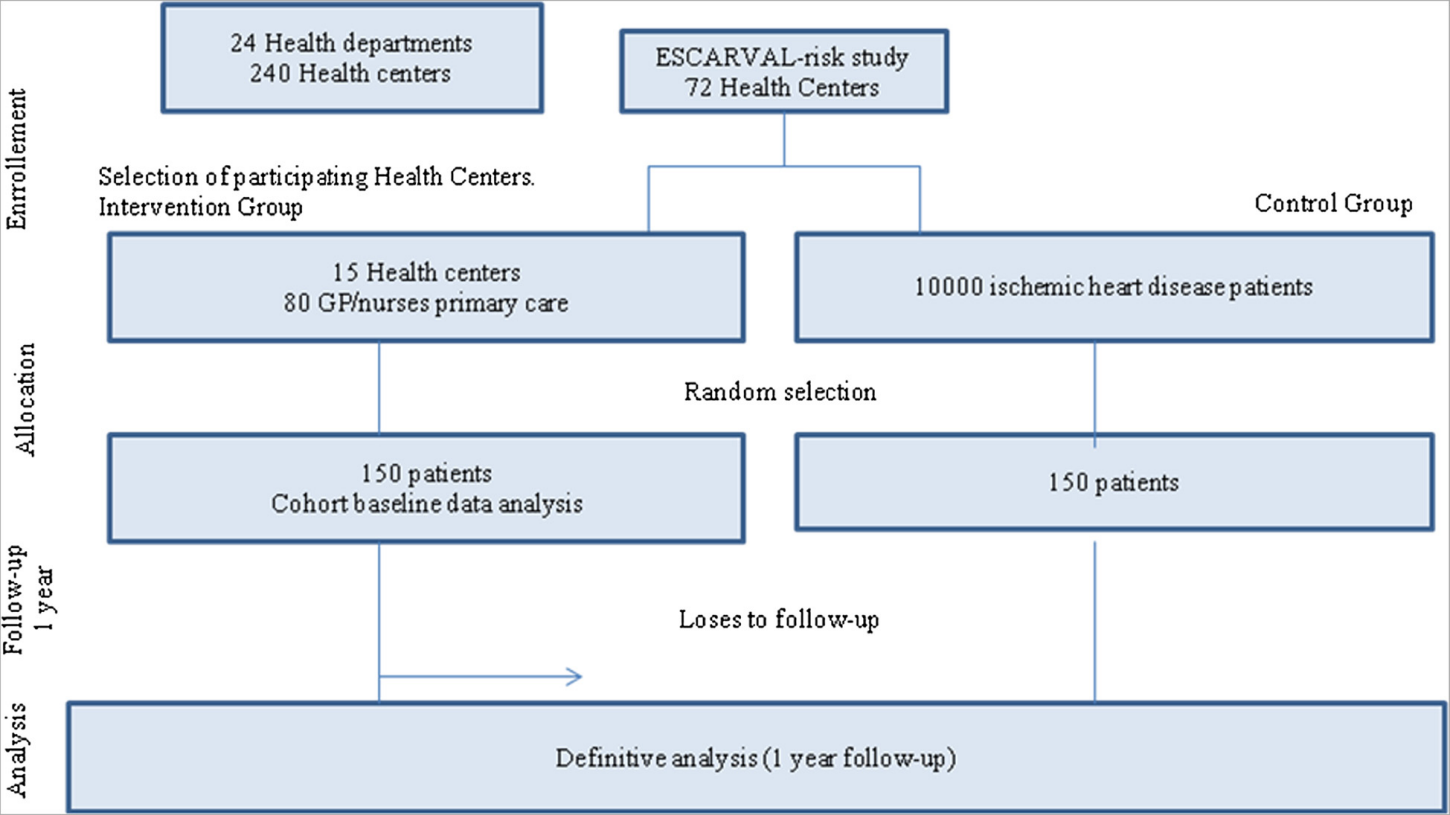
Flow diagram of the PROPRESE study.

Figure 2 provides a flow diagram of the study.

### Ethical and legal issues

This study protocol has been reviewed and approved by the Ethics Committee for Clinical Trials from San Juan de Alicante Hospital (Comite Ético de Investigación Clínica (CEIC) del Hospital Universitario de San Juan de Alicante), on December 2nd, 2009.

The study is conducted according to the standards of the International Guidelines for Ethical Review of Epidemiological Studies (Council for International Organizations of Medical Sciences- CIOMS-Geneva, 1991) and the recommendations of the Spanish Society of Epidemiology about the review of ethical aspects of epidemiological research.

#### Confidentiality of the data

All information relative to the patient’s identity is considered confidential. The data generated during the study will be handled according to the Law 5/1999 and corresponding normative. Any researcher with access to the data used in the study will be required to sign a document guaranteeing confidentiality.

#### Informed consent

All patients must read the “Patient Information Form” and sign a document giving their consent.

## Discussion

IHD is one of the main causes of death despite the great capacity for prevention that is available. Nowadays, multiple therapeutic tools of proven efficacy exist to control the main CVRF, as well as guidelines and protocols endorsed by the main scientific societies [5]–[7,19].

However, the degree of control of the CVRF in patients with IHD is low [15]–[17], which results in a higher use of health services. The frequency of primary care visits of a person with diabetes in Spain is 28 per year, even though this does not necessarily imply greater control [21]. Two of the main reasons for this are lack of treatment compliance and therapeutic inertia in the office, control of which has not improved in recent years [22]–[25].

The CCM [12] consider acting on these factors through new approaches. These models involve patient training to achieve greater autonomy and information, and the identification of high-risk patients to give them a more individualized and protocolized care.

Accordingly, the present study involved an organizational intervention based on CCM together with an educational intervention, integrating cardiology service professionals and primary care professionals (general practitioners and nurses), in an attempt to improve the degree of control of CVRF and decrease the number of hospitalizations.

This intervention aims to increase the degree of self-control for these patients as well as their ability for self-care and decision making to improve their long-term survival. At the same time, the greater degree of information and education can facilitate the increase in compliance that should result in an improvement in the therapeutic objectives.

The health care team-work methodology will be modified with more resolute and specific patient-centered office visits, without increasing the work load in the primary care offices. This is aimed at reducing clinical inertia.

The Abucasis computer system, available in secondary and primary care offices, facilitates the interaction between levels of health care as well as being a common and unique site for the patient to record and control the main CVRF.

The validity of the results, which depends on the representativeness of the sample, is controlled by the selection criteria designed and in relation to study power, through the sample size calculated taking into account any unexpected loss by controlling the random error.

Some multifactorial interventions have been published specially based on nurse work [26] but no very good results have been obtained. Our study has some different aspects that can be mentioned: we use an unique electronic health record for primary and secondary care improving communication between professionals, we implement CCM strategies involving patients with self-care, sharing decisions about the level of control to be reached and objective based treatments, involving all primary care team (physicians and nurses) and cardiologists.

In conclusion, this study will provide information about the efficacy of a patient-centered intervention based on the CCM. Actions will be centered on identifying the higher-risk patients, such as those in secondary prevention; on greater patient information and capacity, favoring autonomy; on shared decision making to establish and reach the control objectives; and an individualized programming of appointments, relying for all this on the teamwork of primary care nurses and physicians under the supervision of the reference cardiology service.

## Abbreviations

IHD: Ischemic heart disease; HC: Health centers; CVD: Cardiovascular disease; CVRF: Cardiovascular risk factors; CCM: Chronic care model; EPP: Expert patient program; LDL: Low density lipoprotein; HDL: High density lipoprotein; SVMFiC: Valencian medical society of family and community medicine. HbA1c: glycated hemoglobin.

## Competing interests

The authors declare they have no competing interests.

## Authors’ contributions

Conception of the idea for the study: DO, ER, MG and AIN. Development of the protocol, organization and funding: DO, ER, MG and AIN. Study design assistance: AC, JN, CC, SP, ES, FB, JB, MAN, VB and VFG. Writing of the manuscript: ER, DO and AIN. All authors have critically read the final manuscript draft, to make contributions, and have approved the final version.

## Pre-publication history

The pre-publication history for this paper can be accessed here:

http://www.biomedcentral.com/1472-6963/13/293/prepub
